# Lifecourse Adversity and Physical Performance across Countries among Men and Women Aged 65-74

**DOI:** 10.1371/journal.pone.0102299

**Published:** 2014-08-07

**Authors:** Ana Carolina Patrício de Albuquerque Sousa, Ricardo Oliveira Guerra, Mai Thanh Tu, Susan P. Phillips, Jack M. Guralnik, Maria-Victoria Zunzunegui

**Affiliations:** 1 CNPQ Programa de Pós Graduação em Ciências da Saúde, Universidade Federal do Rio Grande do Norte, Natal, Brazil; 2 Department of Physiotherapy, Universidade Federal do Rio Grande do Norte, Natal, Brazil; 3 École de Santé Publique, Université de Montréal, Montréal, Canada; 4 Department of Family Medicine, Queens University, Kingston, Canada; 5 Department of Epidemiology and Public Health, Division of Gerontology, University of Maryland School of Medicine, Baltimore, Maryland, United States of America; 6 Centre de Recherche du Centre Hospitalière de l’Université de Montréal (CRCHUM), Montréal, Canada; Cardiff University, United Kingdom

## Abstract

**Background:**

This study examines the associations between lifecourse adversity and physical performance in old age in different societies of North and South America and Europe.

**Methods:**

We used data from the baseline survey of the International Study of Mobility in Aging, conducted in: Kingston (Canada), Saint-Hyacinthe (Canada), Natal (Brazil), Manizales (Colombia) and Tirana (Albania). The study population was composed of community dwelling people between 65 and 74 years of age, recruiting 200 men and 200 women at each site. Physical Performance was assessed with the Short Physical Performance Battery (SPPB). Economic and social adversity was estimated from childhood adverse events, low education, semi-skilled occupations during adulthood and living alone and insufficient income in old age.

**Results:**

A total of 1995 people were assessed. Low physical performance was associated with childhood social and economic adversity, semi-skilled occupations, living alone and insufficient income. Physical performance was lower in participants living in Colombia, Brazil and Albania than in Canada counterparts, despite adjustment for lifecourse adversity, age and sex.

**Conclusions:**

We show evidence of the early origins of social and economic inequalities in physical performance during old age in distinct populations and for the independent and cumulative disadvantage of low socioeconomic status during adulthood and poverty and living alone in later life.

## Introduction

Knowledge of lifecourse conditions and aging across diverse societies is needed to promote healthy aging [1]–[3]. While considerable research describes the relationship between individual circumstances over time, and physical function in older populations [4]–[8] few of these studies include middle and low-income countries where very different childhood and adulthood conditions may shape aging [5]. Most lifecourse studies examine ongoing effects of socioeconomic adversity [3]–[6], [8]–[13]. Newer evidence shows that the strong and latent effects on chronic disease incidence of adverse events occurring in childhood arise from more than economic deprivation. In particular, experiences of childhood violence appear to predict high prevalence of osteoarthritis, heart disease, obesity and headaches in adulthood [14], [15].

After reviewing nineteen studies, *Birnie et al* reported that the associations of childhood socioeconomic position (SEP) with physical performance were maintained after adjustment by adult SEP, suggesting that the accumulation of adverse exposures over a lifetime may be a better model of the associations than one which considers only adult factors and concluded that the associations of childhood socioeconomic position with physical capability vary by study context including geographical location and birth period, whereby SEP in early life may play a more important role in some contexts than others [5].

Studies conducted in European countries, United States, United Kingdom, South America and Korea have focused on social economic position (SEP) to examine associations between lifecourse adversities with physical health outcomes in later life [5]. We identified the need to investigate the relationship of life course and physical function in old age in societies at different stages of socioeconomic development. We propose that early childhood social disadvantage, including exposure to violence and conflict, is an independent risk factor for poor physical function in old age. In addition, following a cumulative disadvantage framework [1], we anticipate that adulthood and old age adversity will have cumulative effects on physical function in old age.

Our research uses baseline data from the International Mobility in Aging Study (IMIAS) to examine associations between lifecourse conditions and physical function in old age in five cities with very different socioeconomic contexts and cultural norms.

The IMIAS is a multicenter and multidisciplinary project, conducted in: Natal (Brazil), Manizales (Colombia), Tirana (Albania), Saint-Hyacinthe (Quebec, Canada) and Kingston (Ontario, Canada). These population differ widely in living conditions and health outcomes, therefore provides an opportunity to examine the associations of life course in old age at different settings.

Natal is a coastal and flat city of 817,590 inhabitants in Northeast Brazil. Manizales is in the coffee-growing zone of the Colombia Andes, with steep terrain (population approximately 400,000). Tirana (Albania), in a central valley, has approximately 700,000 inhabitants. Saint-Hyacinthe is a francophone city (population 50,000), near Montreal, Quebec. Kingston is predominantly Anglophone (population 110,000) and in the province of Ontario. The socioeconomic and cultural characteristics of these cities differ substantially although each is relatively homogeneous, and their elderly populations have resided locally for all or most of their lives.

These sites show considerable variation on the Human Development Index (HDI) and the Gender Inequality Index (GII), published in 2013 by the United Nation Development Program [16]. Probability of survival is very different at these sites as illustrated by life expectancy (LE) for the cohorts born between 1950 and 1954, the earliest period with data provided by the UNDP website. Among those born in 1950–1954, men’s LE was 67 for Canadian and ranged between 49 and 54 for the non-Canadians and women’s LE was 72 for Canadians and ranged between 53 and 56 for non-Canadians.

The IMIAS study provides an important chance to investigate the life course influence on health and function in old people, because it investigates five distinct societies. We aim to identify whether social and economic adversity in early life and during adulthood and old age predict poor physical function in older populations analyzing diverse populations with different social, economic and cultural conditions.

Our research asks whether, among older adults aged 65–74: 1) Does childhood adversity predicts physical performance? 2) Does adulthood adversity predicts physical performance, independent of childhood adversity? 3) Does adversity in old age predicts physical performance independent of earlier adversities in life? This evidence will deepen knowledge on the nature of lifecourse effects on physical function in old age.

## Materials and Methods

### Ethics statement

All of the IMIAS participants gave written informed consent and the study was approved by the ethics committee of each site.

### Study setting

Cross-sectional epidemiological study using baseline data from the IMIAS (International Mobility in Aging Study) project, a population-based prospective cohort study was collected in five sites: Natal (Brazil), Manizales (Colombia), Tirana (Albania), Saint-Hyacinthe (Quebec, Canada) and Kingston (Ontario, Canada).

### Population and Sample

The study population was composed of community-dwelling men and women aged between 65 to 74 years. Stratification by sex occurred at the enrollment stage with the aim of recruiting 200 men and 200 women at each site.

Participants were recruited in 2012 through neighborhood primary care center registers in Tirana, Manizales and Natal. At these sites a random sample of older, community-dwelling adults registered at the neighborhood health center was selected and participants were visited in their homes by our interviewers to invite them to participate. In Kingston and Saint-Hyacinthe, potential participants received a letter from their primary care physicians inviting them to contact our field coordinator. Two different recruitment methods were needed because of Canadian ethics requirements that precluded direct contact with potential participants.

Participants who made 4 or more errors in the orientation scale of the Leganes Cognitive Test (LCT), a screening test for dementia in populations with little education, were excluded [17].

Data collection used assessment tools with known validity among international aging populations. Physical performance tests were conducted at the interview site (usually the participant’s home) everywhere except Manizales, where tests were done at the hospital. Interviewer training was standardized following instructions contained in the IMIAS manual (available upon request). Piloting occurred at each site to estimate response rates and establish logistics of interviews, exams, and blood and saliva collection. A more detailed description of research sites and procedures has been submitted for publication.

#### Outcome

Short Physical Performance Battery (SPPB) is a timed test to assess impairment in gait, balance and muscular strength [18] and is a strong predictor of mobility loss because it captures the hierarchy of functioning from high levels to severe deterioration of lower-extremity function [4], [19]. Poor physical functioning predicts disability, hospitalization, institutionalization and mortality in older populations [20]–[22]. The SPPB includes three hierarchical test of body function: standing balance, a 4-meter walk, and five repetitive chair stands, each SPPB component is scored from 0 (inability) to 4 (high performance). A summary performance score is obtained by adding the scores of each component (range 0–12) with higher scores indicating better lower body function [18]–[20]. For analyses, we used the continuous SPPB score and a dichotomous indicator of low physical performance (SPPB<8) [18], [19], [23].

#### Exposures

Childhood adversity was measured via the following questions on events occurring during the first fifteen years of life: death of parents, abuse of alcohol or drugs by either parent, parental divorce, witnessing physical violence in the family, having been physically abused, low economic status, having been hungry, parent unemployment [5], [7]. Exploratory factor analyses of these indicators with oblimin rotation yielded three underlying factors. The first was named social adversity and included: parental alcohol or drug abuse, witnessing family physical violence and having been physically abused. The second represented economic adversity and included poor economic status, hunger, and parental unemployment. The third factor was composed of death or divorce of parents. Since the corresponding eigenvalue was barely 1 and Cattell’s scree test indicated a break we did not take into account this factor. The percentage of explained common variance using the first two factors was 54%. Correlation between the two factors was 22%. Then, two scores were calculated, social and economic adversity, by counting the frequency of events for the contributing indicators. Thus, economic and social adversity ranged from 0 to 3.

Adversity in adulthood was indicated by less than secondary education (compared to secondary or post secondary education) and semi-skilled occupation (compared with skilled and non-manual occupation). The nature of longest held occupation was measured with open questions and recoded according to the International Standard Classification of Occupations in 10 major groups then further classified into three groups: 1. Non manual; 2. Skilled manual: machine operators and assemblers and crafts and related trades; 3. Semi-skilled manual: farm work, services, elementary occupations or housewife.

Adversity in old age was indicated by insufficiency of monthly income for basic needs (compared to very sufficient and sufficient income) and living alone (compared to living only with partner or with children, spouse and/or others).

Potential confounders considered were: age, sex, research site.

### Statistical Analysis

Data were analyzed using SPSS (Statistical Package for the Social Sciences) 20.0. Bivariate analyses were as performed via Pearson’s chi square (Χ^2^) test to identify a possible association between life course adversity and low physical performance (SPPB<8). Using ANOVA, means of SPPB were compared across indicators of life course adversity.

First, logistic regressions were fitted to examine the associations of lifecourse adversity with low physical performance using staggered entry of variables with the following order: childhood, adulthood and old age. Every model was adjusted by sex, age and site. Second, site-specific regressions were carried out to assess the independent contributions of childhood, adulthood and old age adversity to low physical performance at each site. Odds ratios for logistic models were estimated with 95%CI.

Lastly, taking account the possible differences in associations between countries, we have conducted a meta-analyses to estimate adversity indicators overall effects on SPPB based on the five distinct studies using identical methods.

## Results

All 1995 participants were born between 1938 and 1947 with mean age of 69.1±2.9 years and 55.2% between ages 65 to 69. Women made up 52.1% of the overall sample. Exclusion due severe cognitive impairment varied between 0–2% by site.

The distributions of lifecourse adversity are shown in Table 1. Early social and economic adversity varied significantly by site (p<0.001). In Natal 26.6% of the elderly participants reported one or more adverse social conditions in childhood, followed by Kingston (25.6%), Saint-Hyacinthe (24.9%), Manizales (23.5%) and Tirana (17.8%). A high proportion of participants experienced early economic adversity, with site-specific proportions being: Natal (64.9%), Tirana (57.4%), Manizales (41.8%), Saint-Hyacinthe (35.7%) and Kingston (31.9%).

**Table 1:** Lifecourse Adversity By Research Site.

	NATAL (402)	MANIZALES (400)	TIRANA (394)	SAINT- HYACINTHE (401)	KINGSTON (398)	p-value
**CHILDHOOD SOCIAL ADVERSITY** (%)						
**Parental abuse of alcohol/drugs**	15.7	9.8	4.3	12.2	17.1	0.001
**Witnessing family physical violence**	14.7	9.0	14.0	15.0	12.1	0.069
**Physically abused**	6.5	12.0	11.4	9.2	12.1	0.039
**SCORE SOCIAL** a						
**0**	73.4	76.5	82.2	75.1	74.4	0.001
**1**	17.9	17.5	7.4	15.7	13.8	
**2**	7.2	4.8	8.9	7.0	8.0	
**3**	1.5	1.2	1.5	2.2	3.8	
**CHILDHOOD ECONOMIC ADVERSITY** (%)						
**Early economic**						
Good or Average	40.3	62.8	59.1	70.6	73.9	0.001
Poor	59.7	37.2	40.9	29.4	26.1	
**Parent unemployment**	21.1	11.8	15.2	7.7	9.0	0.001
**Hunger**	34.8	11.8	41.4	4.0	6.0	0.001
**SCORE ECONOMIC** b						
**0**	35.1	58.2	42.6	64.3	68.1	0.001
**1**	28.1	28.5	26.1	30.7	23.4	
**2**	22.9	7.5	22.3	4.5	7.8	
**3**	13.9	5.8	8.9	0.5	0.8	
**ADULTHOOD ADVERSITY** (%)						
**Education**						
Less Secondary	80.6	71.5	9.9	17.0	3.5	0.001
Secondary	15.2	17.0	27.9	32.4	18.8	
Post Secondary	4.2	11.5	62.2	50.6	77.6	
**Occupation**						
No Manual	10.4	15.5	36.3	48.6	75.9	0.001
						
Skilled manual	44.8	43.8	56.3	26.2	9.8	
Semi-Skilled manual	44.8	40.8	7.4	25.2	14.3	
**OLD AGE ADVERSITY** (%)						
**Income**						
Very sufficient	4.0	4.8	37.6	44.1	61.1	0.001
Sufficient	21.9	24.0	41.4	48.4	33.7	
Insufficient	74.1	71.2	21.1	7.5	5.3	
**Living Arrangements**						
Alone	6.5	14.2	9.9	26.2	31.7	0.001
Only Spouse	19.2	18.0	44.7	65.8	45.0	
Children, spouse or others	74.4	67.8	45.4	8.0	23.4	

a indicators that loaded on social adversity were: abuse of alcohol/drugs, witnessing violence and experiencing physical abuse (factor analyses).

b indicators that loaded on economic adversity were: low economic situation, hunger, parent unemployment (factor analyses).

Indicators of adulthood and current adversity also varied significantly across sites. Natal had the lowest education and the highest proportion of income insufficiency. Kingston and Tirana had highest education, whereas in Saint-Hyacinthe and Kingston the participants reported the highest proportions of sufficiency of income. Living arrangements vary with higher proportions of older participants living alone in the Canadian sites.

The prevalence of low physical performance (SPPB<8) was greatest in Natal (Table 2). Using comparisons of mean SPPB scores, poorer performance was found among non-Canadian than in Canadian sites.

**Table 2:** Short Physical Performance Battery by Site and Lifecourse Adversity Indicators.

	SPPB<8	SPPB 8 to 10	SPPB 11 and 12	p-value	SPPB TOTAL Mean ± SD	p-value
**SITE**				0.001		0.001
Natal	22.4	44.4	33.6		9.09±2.407	
Manizales	18.5	68.2	13.2		8.85±1.679	
Tirana	20.8	43.4	35.8		9.05±2.802	
Saint-Hyacinthe	6.7	45.4	47.9		10.18±1.630	
Kingston	7.8	35.4	56.8		10.28±1.851	
Childhood adversity						
**Parental abuse of alcohol/drugs**				0.053		0.120
YES	18.2	51.3	30.5		9.28±2.151	
NO	14.8	46.8	38.4		9.52±2.212	
**Witness physical violence**				0.001		0.001
YES	22.1	47.7	30.2		9.06±2.467	
NO	14.2	47.3	38.5		9.55±2.158	
**Physically abused**				0.004		0.001
YES	23.0	44.1	32.8		8.99±2.531	
NO	14.3	47.7	38.0		9.55±2.159	
**Social Adversity score^a^**				0.001		0.001
0	14.3	46.7	39.0		9.56±2.174	
1	14.2	50.5	35.3		9.52±2.052	
2	22.4	49.0	28.7		8.94±2.622	
3	34.1	39.0	26.8		8.56±2.419	
**Family economic situation**				0.002		0.001
Good or Average	13.3	46.9	39.8		9.62±2.191	
Poor	18.3	48.1	33.7		9.28±2.214	
**Parent unemployment**				0.002		0.008
YES	22.4	45.6	32.0		9.15±2.315	
NO	14.2	47.6	38.2		9.54±2.185	
**Hunger**				0.001		0.001
YES	26.2	44.9	29.0		8.82±2.533	
NO	12.6	47.9	39.5		9.65±2.087	
**Economic adversity score^b^**				0.001		0.001
0	11.9	47.5	40.6		9.70±2.114	
1	15.6	47.8	36.6		9.48±2.126	
2	23.2	45.9	30.9		8.93±2.579	
3	26.9	46.2	26.9		8.88±2.195	
Adulthood adversity						
**Education**				0.001		0.001
Less Secondary	20.9	55.1	23.9		8.93±2.141	
Secondary	15.8	50.2	34.0		9.42±2.290	
Post Secondary	9.9	38.8	51.3		10.03±2.085	
**Longest held Occupation**				0.001		0.001
No Manual	9.5	41.1	49.3		10.02±2.044	
Skilled manual	17.6	50.1	32.3		9.21±2.318	
Semi-Skilled manual	20.0	52.3	27.7		9.13±2.127	
Old age adversity						
**Income**				0.001		0.001
Very sufficient	7.8	40.6	51.6		10.13±1.913	
Sufficient	13.2	47.6	39.3		9.61±2.134	
Insufficient	23.4	52.7	23.8		8.84±2.325	
**Living Arrangements**				0.001		0.001
Alone	17.0	45.0	38.0		9.49±2.220	
Only Spouse	9.8	42.4	47.8		9.94±2.113	
Children and others	19.3	52.5	28.1		9.10±2.207	

As hypothesized, greater childhood social and economic adversity was associated with low physical performance (p<0.001). Adulthood and old age adversity showed similar associations (p<0.001).

Multivariate models were fitted according to exposure to adversity in childhood, adulthood and old age. Three models addressed our research questions and all controlled for sex, age and site (Tables 3, 4, 5). The first column of these tables shows the models using the whole data. Table 3 shows results for childhood adversity (model 1). Both social and economic adversity was independently associated with low physical performance. In Table 4 (model 2), semi-skilled manual labor during adulthood was associated with low physical performance independent of childhood adversity. Lastly, in Table 5 (model 3), living alone and having insufficient income to cover basic needs in old age were associated with low physical performance independent of childhood or adult adversity. In all models, those living in Tirana, Manizales and Natal (compared to Kingston) had elevated odds ratios for poor physical function. Those living in Saint-Hyacinthe were not different from those living in Kingston. Women had higher odds of poor physical function compared with men and poor physical function was associated with age.

**Table 3:** Associations between Childhood Adversity and Poor Physical Performance (SPPB<8).

VARIABLES	ALL SITES	NATAL	MANIZALES	TIRANA	SAINT-HYACINTHE	KINGSTON
MODEL 1: OR [CI 95%] for CHIDHOOD ADVERSITY + SEX, AGE, SITE						
**Social Adversity** a						
0	1.00	1.00	1.00	1.00	1.00	1.00
1	0.918[0.627–1.344]	0.822[0.436–1.551]	0.487[0.214–1.108]	0.367[0.082–1.652]	**4.073** **[1.573–10.542]**	1.573[0.548–4.512]
2	1.562[0.998–2.443]	0.582[0.227–1.493]	1.546[0.593–4.031]	**4.380** **[2.050–9.360]**	**4.303** **[1.383–13.393]**	2.392[0.825–6.932]
3	**3.359** **[1.644–6.863]**					
**Economic Adversity** a						
0	1.00	1.00	1.00	1.00	1.00	1.00
1	1.268[0.931–1.728]	0.996[0.540–1.840]	1.613[0.899–2.893]	1.163[0.571–2.367]	1.911[0.792–4.611]	0.997[0.408–2.438]
2	**1.608** **[1.107–2.336]**	1.230[0.698–2.168]	1.275[0.567–2.868]	**2.714** **[1.448–5.088]**	1.425[0.326–6.238]	0.872[0.226–3.374]
3	1.628[0.999–2.652]					
**Sex**						
Male	1.00	1.00	1.00	1.00	1.00	1.00
Female	**2.003** **[1.537–2.609]**	**1.976** **[1.208–3.233]**	**1.835** **[1.076–3.131]**	**3.986** **[2.192–7.247]**	1.806[0.755–4.318]	1.163[0.545–2.485]
**Age**	**1.093** **[1.046–1.142]**	**1.072** **[0.981–1.172]**	**1.104** **[1.011–1.206]**	1.059[0.971–1.154]	**1.182** **[1.014–1.377]**	**1.160** **[1.009–1.332]**
**Site**						
Kingston	1.00					
Saint-Hyacinthe	0.927[0.538–1.598]					
Tirana	**2.922** **[1.845–4.627]**					
Manizales	**2.871** **[1.816–4.538]**					
Natal	**3.217** **[2.032–5.093]**					

a **Scores Social and Economic: 0(none); 1(one); 2(two or more) for site-specific analyses.**

**Table 4:** Associations between Adulthood Adversity and Poor Physical Performance (SPPB<8).

VARIABLES	ALL SITES	NATAL	MANIZALES	TIRANA	SAINT-HYACINTHE	KINGSTON
MODEL 2: OR [CI 95%] for ADULTHOOD ADVERSITY + CHIDHOOD + SEX, AGE, SITE						
**Education**						
Post Secondary	1.00	1.00	1.00	1.00	1.00	1.00
Secondary	1.266 [0.787–2.037]	0.429 [0.129–1.433]	0.931 [0.319–2.714]	1.152 [0.451–2.941]	1.585 [0.450–5.564]	0.882 [0.155–5.002]
Less secondary	1.299 [0.868–1.943]	0.320 [0.090–1.130]	0.675 [0.209–2.179]	1.473 [0.734–2.958]	1.253 [0.429–3.664]	1.161 [0.459–2.933]
**Occupation**						
No Manual	1.00	1.00	1.00	1.00	1.00	1.00
						
Skilled manual	1.191 [0.801–1.771]	0.722 [0.272–1.921]	0.994 [0.385–2.569]	1.031 [0.523–2.032]	1.258 [0.320–4.949]	**3.906 [1.330–11.468]**
Semi-Skilled manual	**1.558 [1.039–2.336]**	1.256 [0.498–3.168]	1.125 [0.443–2.859]	**0.156 [0.029–0.849]**	**3.816 [1.239–11.749]**	1.184 [0.688–5.157]
**Childhood Social Adversity**						
0	1.00	1.00	1.00	1.00	1.00	1.00
1	0.907 [0.618–1.329]	0.837 [0.441–1.589]	0.473 [0.207–1.084]	0.358 [0.078–1.648]	**3.696 [1.383–9.876]**	1.450 [0.495–4.245]
2	1.530 [0.976–2.400]	0.608 [0.235–1.572]	1.514 [0.571–4.017]	**5.021 [2.233–11.289]**	**3.874 [1.190–12.616]**	2.717 [0.907–8.143]
3	**3.311 [1.613–6.779**]					
**Childhood Economic** **Adversity**						
0	1.00	1.00	1.00	1.00	1.00	1.00
1	1.263 [0.925–1.725]	1.002 [0.538–1.864]	1.581 [0.869–2.875]	1.288 [0.625–2.654]	1.824 [0.735–4.523]	0.963 [0.393–2.363]
2	**1.606 [1.099–2.345]**	1.349 [0.756–2.406]	1.230 [0.541–2.797]	**2.809 [1.473–5.357]**	1.217 [0.262–5.647]	0.940 [0.233–3.796]
3	1.545 [0.945–2.527]					
**Sex**						
Male	1.00	1.00	1.00	1.00	1.00	1.00
Female	**1.914 [1.459–2.511]**	**1.846 [1.101–3.095]**	**1.828 [1.046–3.194]**	3.873 [2.060–7.281]	1.728 [0.699–4.271]	1.321 [0.604–2.889]
**Age**	1.090 [1.043–1.140]	1.069 [0.976–1.172]	**1.104 [1.011–1.206]**	1.046 [0.957–1.142]	1.142 [0.971–1.343]	**1.163 [1.011–1.337]**
**Site**						
Kingston	1.00					
Saint-Hyacinthe	0.780 [0.444–1.369]					
Tirana	**2.674 [1.647–4.343]**					
Manizales	**2.046 [1.180–3.547]**					
Natal	**2.243 [1.279–3.934]**					

**Table 5:** Associations between Old Age Adversity and Poor Physical Performance (SPPB<8).

VARIABLES	ALL SITES	NATAL	MANIZALES	TIRANA	SAINT-HYACINTHE	KINGSTON
MODEL 3: OR [CI 95%] for OLD AGE + ADULTHOOD ADVERSITY + CHIDHOOD + SEX, AGE, SITE						
**Living Arrangements**						
Only Spouse	1.00	1.00	1.00	1.00	1.00	1.00
Alone	**1.774** **[1.191–2.642]**	1.214[0.406–3.630]	1.244[0.459–3.374]	0.919[0.353–2.390]	**4.934** **[1.767–13.779]**	**3.182** **[1.241–8.162]**
Children and others	1.350[0.967–1.884]	1.145[0.594–2.207]	1.375[0.641–2.951]	1.163[0.648–2.087]	3.224[0.596–17.432]	1.655[0.526–5.203]
**Income**						
Very sufficient	1.00	1.00	1.00	1.00	1.00	1.00
Sufficient	1.413[0.948–2.106]	1.286[0.308–5.374]	0.590[0.140–2.482]	1.196[0.619–2.310]	1.066[0.371–3.068]	1.707[0.735–3.965]
Insufficient	**2.174** **[1.394–3.389]**	1.392[0.355–5.459]	1.383[0.374–5.123]	1.642[0.779–3.461]	2.577[0.598–11.102]	**4.644** **[1.278–16.874]**
**Childhood Social** **Adversity**						
0	1.00	1.00	1.00	1.00	1.00	1.00
1	0.893[0.608–1.311]	0.837[0.441–1.591]	0.481[0.209–1.106]	0.368[0.080–1.694]	3.465[1.222–9.829]	1.739[0.574–5.267]
2	1.438[0.911–2.269]	0.616[0.238–1.595]	1.574[0.582–4.256]	**4.693** **[2.032–10.842]**	2.610[0.732–9.303]	2.689[0.852–8.481]
3	**3.067** **[1.474–6.383]**					
**Childhood Economic** **Adversity**						
0	1.00	1.00	1.00	1.00	1.00	1.00
1	1.220[0.890–1.672]	1.000[0.537–1.865]	1.548[0.845–2.837]	1.240[0.597–2.574]	1.676[0.635–4.423]	0.911[0.357–2.324]
2	**1.533** **[1.046–2.246]**	1.330[0.741–2.388]	1.095[0.475–2.524]	**2.649** **[1.378–5.094]**	1.435[0.272–7.565]	0.674[0.151–2.996]
3	1.443[0.877–2.375]					
**Education**						
Post Secondary	1.00	1.00	1.00	1.00	1.00	1.00
Secondary	1.134[0.701–1.836]	0.400[0.115–1.388]	0.756[0.254–2.254]	1.129[0.441–2.893]	1.027[0.263–4.007]	0.796[0.138–4.599]
Less secondary	1.211[0.805–1.821]	0.303[0.084–1.097]	0.615[0.188–2.012]	1.403[0.694–2.834]	1.094[0.355–3.374]	1.154[0.442–3.017]
**Longest held Occupation**						
No Manual	1.00	1.00	1.00	1.00	1.00	1.00
						
Skilled manual	1.127[0.755–1.681]	0.712[0.266–1.904]	0.865[0.328–2.279]	0.997[0.500–1.989]	1.026[0.239–4.406]	2.942[0.940–9.215]
Semi-Skilledmanual	**1.516** **[1.006–2.284]**	1.244[0.492–3.149]	1.004[0.389–2.589]	**0.149** **[0.027–0.825]**	**3.988** **[1.214–13.100]**	1.863[0.661–5.247]
**Sex**						
Male	1.00	1.00	1.00	1.00	1.00	1.00
Female	**1.738** **[1.319–2.290]**	**1.800** **[1.065–3.043]**	**1.788** **[1.016–3.144]**	**3.695** **[1.929–7.076]**	1.035[0.367–2.920]	1.041[0.445–2.433]
**Age**	**1.093** **[1.045–1.144]**	1.701[0.977–1.175]	**1.110** **[1.014–1.214]**	1.049[0.960–1.147]	1.119[0.944–1.325]	**1.164** **[1.004–1.349]**
**Site**						
Kingston	1.00					
Saint-Hyacinthe	0.793[0.446–1.412]					
Tirana	**2.590** **[1.564–4.289]**					
Manizales	**1.402** **[0.763–2.575]**					
Natal	**1.597** **[0.858–2.974]**					

The second to fifth columns (Tables 3, 4 and 5) show site-specific results of the three multivariate models. Small sample sizes lead to wide and statistically insignificant confidence intervals generally, but a pattern of attenuation of significant associations between lifecourse adversity and poor function was observed for Latin American sites whereas in Tirana, associations between childhood social and economic adversities and poor physical performance remained significant in all models (Table 3). A very strong association between childhood social adversity and poor physical function is observed in Saint-Hyacinthe, while in Kingston the odd ratios are suggestive of important associations but with wide confidence intervals (Table 3). Associations between adulthood adversity indicators and poor function were attenuated or became inconsistent in the non-Canadian sites. In Kingston and Saint-Hyacinthe, skilled or semi-skilled manual occupations were associated with poor physical function (Table 4). Lastly, adversity in old age was associated with poor physical function in the Canadian sites where both insufficient income and living alone emerge as risk factors for poor physical function after controlling for earlier lifecourse adversity (Table 5).

In the meta-analyses, comparing subjects who had experienced childhood social adversity in childhood with those that had not, and adjusting for childhood economic adversity, age and sex, the pooled odds ratio was OR = 2.02 (95%CI: 1.00; 4.11; p-heterogeneity = 0.02); the corresponding figures for childhood economic adversity were OR = 1.43 (95%CI: 0.95; 2.15; p-heterogeneity = 0.21). The overall association of low education with low SPPB, adjusting for childhood disadvantage, age and sex was not significant OR = 1.25 (95%CI: 0.75; 1.92; p-heterogeneity = 0.15). The overall association of manual occupation with low SPPB was not significant OR = 1.37 (95%CI: 0.88; 2.16; p-heterogeneity = 0.241). Lastly, income insufficiency had an overall effect of OR = 1.55 (95%CI 1.06; 2.28; p-heterogeneity = 0.895) and living alone or with other than the spouse had an overall OR = 1.59 (95%CI: 1.02; 2.49; p-heterogeneity = 0.10). See Figure S1 to Figure S6 in File S1.

## Discussion

We explored whether low physical performance in old age is related to exposure to social and economic adversity during the lifecourse and how that relationship varies across societies. Although SES in early life has been previously associated with adverse health and functional outcomes in later life, including poor physical performance [4]–[6], [8], [9], few studies have examined the associations between social adversity and physical performance in old age.

Both childhood social and economic adversity independently predicted lower physical performance decades later. Previous research has shown that economic disadvantage in childhood is associated with a range of adverse outcomes in adulthood, often independent of adulthood socioeconomic status [8], [10], [24]–[26], suggesting that apparent current effects of economic adversity on health outcomes [5] may be a proxy for an accumulation of adverse exposures over a lifetime [6]. In addition to the effects of childhood economic adversity, we found a significant and graded association between childhood social adversity and poor physical performance, strongest in the most advantaged of our study sites, i.e. those in Canada. Social adversity indicators as physical abuse and violence may have been under-reported in Brazil and Colombia where tolerance and norms for violence are higher. These Latin American countries historically have higher frequencies of violence and particularly domestic violence than does Canada, as documented by the World Health Organization [27].

Both income and education have been studied extensively in the literature on health inequalities in older populations. Occupation is often the most common SES measure used as the basis for social class comparisons in studies conducted in the United Kingdom [13], [28], [29]. A combination of these three indicators has been recommended to assess the effect of socioeconomic status on the health of aging populations [11], [13], [30].

In our study, semi-skilled occupations and insufficient income were associated with poor physical performance independent of childhood adversity and after adjustment for sex, age and site. However, education was not independently associated with physical performance, suggesting that occupation and income are the stronger socioeconomic determinants of function in the studied populations. An alternative explanation could be that education categories were too wide to assess whether differences exist. The vast majority of Latin American participants had less than secondary education, and this category includes those that are illiterate, who have completed primary school and those having initiated secondary school. These wide categories are appropriate for cross-country analyses; however we acknowledge their SES meaning is not equivalent across sites.

Living alone was an independent risk factor for low physical function among our populations. Living alone may be a proxy for weak social networks and it is widely accepted that social networks protect for physical function in old age [31], [32]. In our site-specific analyses, living alone emerges as an important predictor of poor function only in Canada. Again, comparisons across sites are difficult since in Latin America and Albania disabled elderly people often live with children precisely as a consequence of disability. Thus, in these populations reverse causality is likely since it is functional decline that leads to the change from living alone or with a spouse to living with children or other family members. Notice that in the meta-analyses we have compared living alone and living with someone other than the spouse with all those that live only with the spouse, to take into account these societal differences in living arrangements.

Independent of lifecourse adversities, there were specific effects of the research site and sex on functional outcomes in old age, which reflect differences in conditions of aging between countries with high socioeconomic inequalities or a former communist economy, in health and social protection systems, in human development and in gender equality. More international research on conditions for active aging is needed to better understand these specific gender and site effects. Several studies on lifecourse adversity and physical function in old age have been conducted in Europe, USA, UK, South America and Korea [5], but few include multiple sites to enable comparisons across diverse populations and social norms [5]. The multinational IMIAS cohort provides an opportunity to investigate the influence of lifecourse conditions on health and function in a narrow older age group while also considering wide ranging exposures and lived environments. Additional strengths of this work are the rigorous study design, the use of validated methods and the excellent performance of the well-trained data collectors.

Nevertheless, there are some potential sources of bias and lack of precision. Recruitment procedures in Canada were indirect, as required by ethics review boards, with a response rate of 30% of the original target sample. A comparison of the study samples with the 2006 Canadian census shows that the percentages of the population with college education in the 65 to 74 age group were 55% (Kingston) and 46% (Saint-Hyacinthe). Thus, participants in our Kingston cohort have higher education than the reference population surveyed in the 2006 census, whereas differences were smaller in Saint-Hyacinthe. This suggests that our findings regarding physical function and adversity in Kingston may give an overoptimistic view of the reality of older adults living in Kingston.

As mentioned before, life expectancy at birth for a cohort born in 1950–1954 varied across sites, suggesting that those surviving to age 65 and able to participate in IMIAS in Natal, Manizales and Tirana belong to the fittest of their birth cohorts. Differential survival might arise from variation in childhood adversity and may produce an underestimate of the effect of social and economic adversity on physical function in these sites. This mortality selection may explain the attenuated associations between social and economic adversity and low physical performance in the non-Canadian study sites. In addition, under-reporting of adversity exposure may be more frequent in non-Canadian sites where poverty and violence are more widespread and tolerated. This argument is supported by our finding of similar frequencies in reporting hunger and violence across research sites.

Confidence intervals for the odds ratio estimated in site-specific analyses are sometimes wide leading to imprecision. Sample size at each research site (n = 400) may be insufficient to test associations in models with a large number of co-variables. Lastly, 25 people from Manizales had missing SPPB scores and were, therefore, excluded from analyses, however, the difference in self-reported mobility between those 25 people and the remainder of the Manizales sample was not significant.

Despite these limitations, meta-analysis results show consistency of associations across societies, although there is some heterogeneity that could be related to stronger relationships in Canadian sites due to the differential survival of those subject to early adversity and particularly to their lower survival in the Latin American contexts.

## Conclusion

This study confirms the independent and cumulative associations of lifecourse adversity at each stage of the lifecourse with physical function in older adults. We provide new evidence for the independent effects of childhood experiences of conflict and violence, beyond the already known effects of childhood poverty, on physical function in old age across several distinct populations. This research supports the increasing body of literature showing that early social and economic inequalities continue to do harm years later and also, the cumulative disadvantage in physical function among those exposed to social and economic adversity in adulthood and old age. Promotion of healthy aging requires considering policies and practices that increase social and economic wellbeing for children and youth as well as adults, as early adversity has an ongoing and negative effect well into old age.

## Supporting Information

File S1 **This file contains Figures S1–S6.** Figure S1, Odds ratio for childhood social adversity adjusting for childhood economic adversity, age and sex. Figure S2, Odds ratio for childhood economic adversity adjusting for childhood social adversity, age and sex. Figure S3, Odds ratio for income adjusting for childhood adversity, education, living arrangement, age and sex. Figure S4, Odds ratio for living alone or with someone other than the spouse, adjusting for childhood adversity, education, insufficient income, age and sex. Figure S5, Odds ratio for secondary education adjusting for childhood adversity, age and sex. Figure S6, Odds ratio for manual occupation adjusting for childhood adversity, education, age and sex.
(DOCX)
