# Supplementary material for: Lifecourse Adversity and Physical Performance across Countries among Men and Women Aged 65-74
Source: PLoS One. 2014 Aug 7;9(8):e102299. doi: 10.1371/journal.pone.0102299 (PMC4125146; doi:10.1371/journal.pone.0102299)

Figure S1: Odds ratio for childhood social adversity adjusting for childhood economic adversity, age and sex.


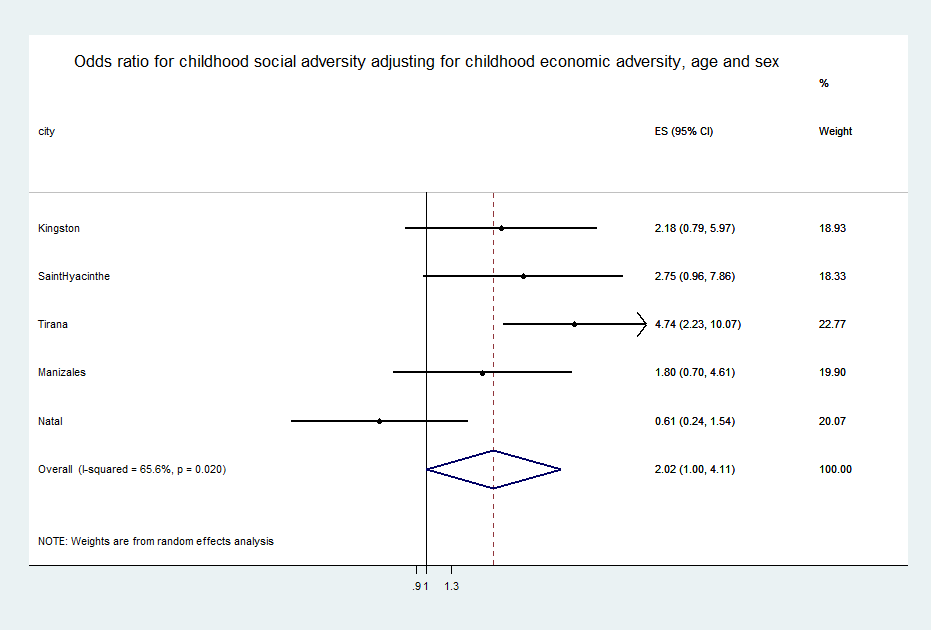


Figure S2: Odds ratio for childhood economic adversity adjusting for childhood social adversity, age and sex.


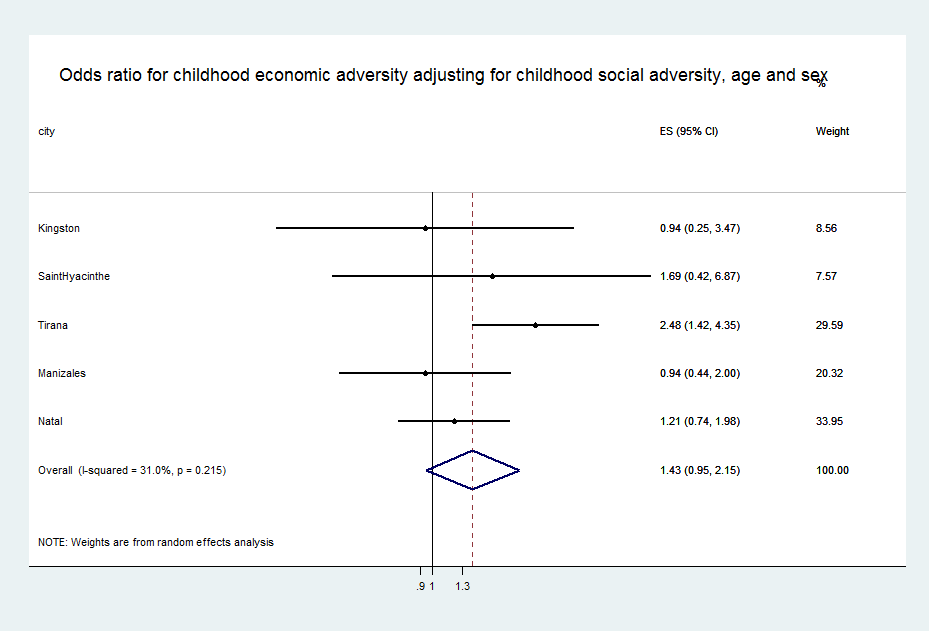


Figure S3: Odds ratio for income adjusting for childhood adversity, education, living arrangement, age and sex.


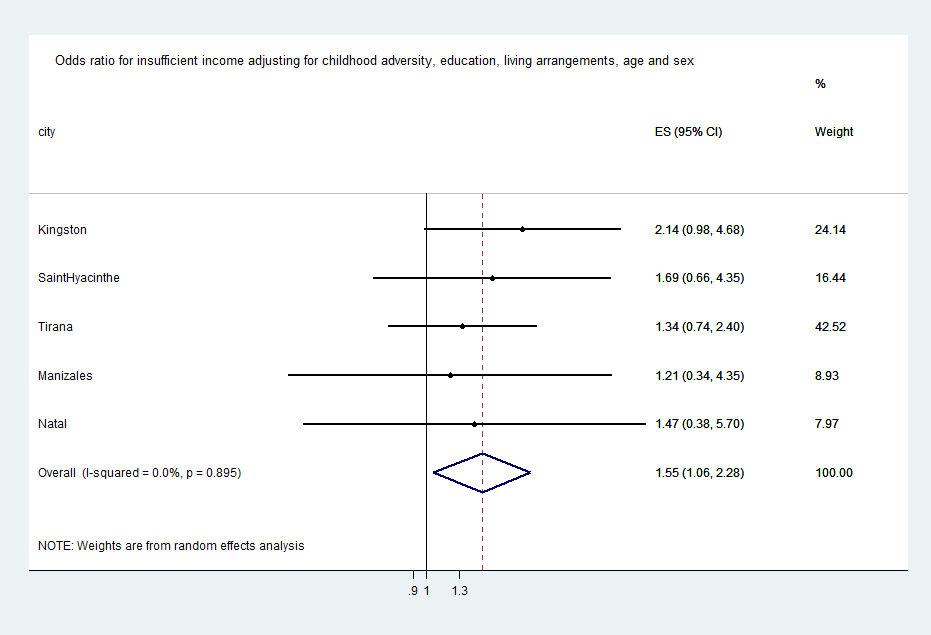


Figure S4: Odds ratio for living alone or with someone other than the spouse, adjusting for childhood adversity, education, insufficient income, age and sex.


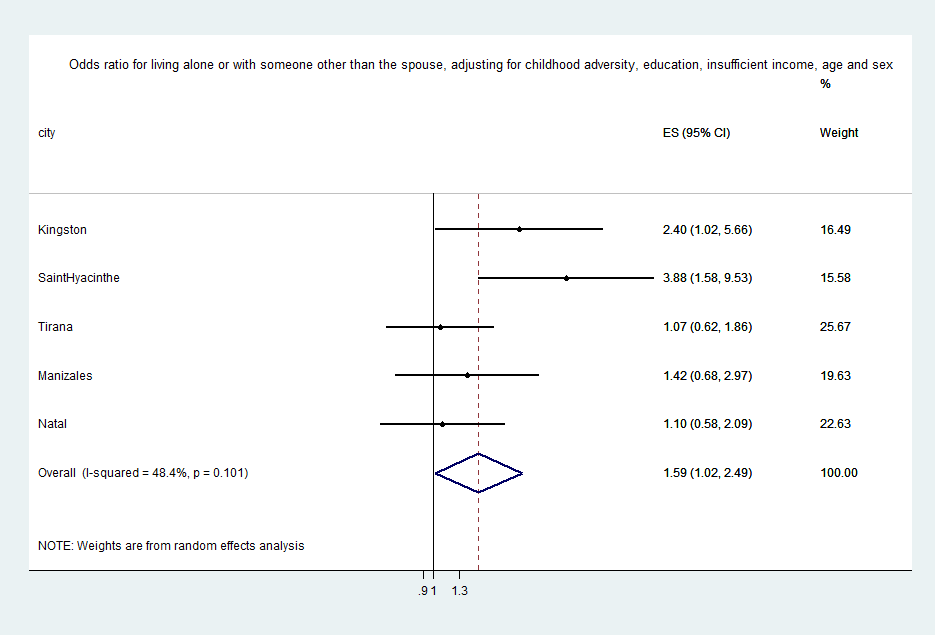


Figure S5: Odds ratio for secondary education adjusting for childhood adversity, age and sex.


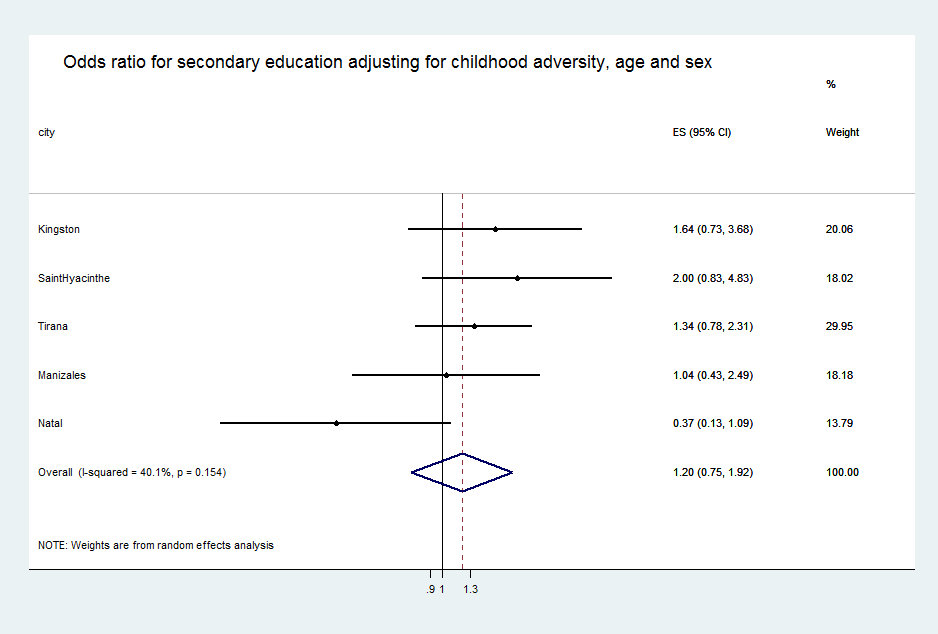


Figure S6: Odds ratio for manual occupation adjusting for childhood adversity, education, age and sex.


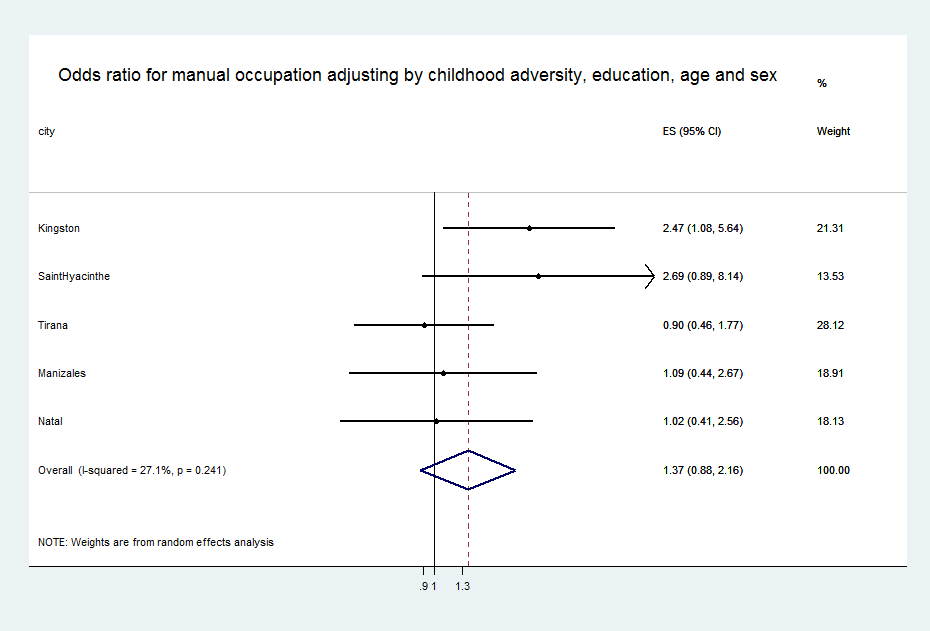

Supplement: File S1 — This file contains Figures S1–S6. Figure S1, Odds ratio for childhood social adversity adjusting for childhood economic adversity, age and sex. Figure S2, Odds ratio for childhood economic adversity adjusting for childhood social adversity, age and sex. Figure S3, Odds ratio for income adjusting for childhood adversity, education, living arrangement, age and sex. Figure S4, Odds ratio for living alone or with someone other than the spouse, adjusting for childhood adversity, education, insufficient income, age and sex. Figure S5, Odds ratio for secondary education adjusting for childhood adversity, age and sex. Figure S6, Odds ratio for manual occupation adjusting for childhood adversity, education, age and sex. (DOCX) [file pone.0102299.s001.docx]
